# Attitudes of Jordanians Toward Therapeutic Abortion—A Qualitative Study

**DOI:** 10.3390/healthcare13182263

**Published:** 2025-09-10

**Authors:** Roqia S. Maabreh, Hekmat Y. Al-Akash, Mohammad N. Al-Shloul, Naser A. Alsharairi

**Affiliations:** 1 Nursing College, Irbid National University, Irbid 21110, Jordan; r.maabreh@inu.edu.jo; 2 Faculty of Nursing, Applied Science Private University, Amman 11937, Jordan; hikmat.akash@gmail.com; 3 Prince Al Hussein Bin Abdullah II Academy for Civil Protection, Al-Balqa’ Applied University, Amman 11183, Jordan; dralshloulm@gmail.com; 4 Heart, Mind and Body Research Group, Griffith University, Gold Coast, QLD 4222, Australia

**Keywords:** therapeutic abortion, Jordanians, attitudes, qualitative study

## Abstract

**Background**: Therapeutic abortion is a controversial topic since people throughout the world have different views on it. **Methods**: In this qualitative study, 12 adults (ages 19–36 years) from Irbid, in the Northern region of Jordan, were selected from a variety of public locations (markets, mosques) in April and May 2025 to participate in semi-structured interviews with the goal of exploring attitudes toward therapeutic abortion. The interviews, which lasted an average of 30 min, focused on two primary topics: attitudes about therapeutic abortion and determining who has the authority to make abortion decisions. An audio recorder was used to capture the responses, which were then preserved in their original, unaltered state. Following verbatim transcription, the responses were subjected to thematic analysis in order to determine the main themes. The original code was made manually. **Results**: Three themes were identified under the first topic: “unconditional rejection”, “conditional acceptance”, and “empathetic and woman-centered attitudes”. Medical and religious experts’ decision-making arose by the theme of “abortion decision” that emerged in relation to the second topic. **Conclusions**: Jordanians have conflicting views on therapeutic abortion and are adamantly opposed to making their own decisions on the matter.

## 1. Introduction

The Global Burden of Disease Study (GBD) reports that the number of abortions and miscarriages decreased from 49 million in 1990 to 42 million in 2019. Some countries still have a significant disease burden despite this drop. For example, Ethiopia had the highest age-standardized incidence rate in 2019 at 3839.06 per 100,000, followed by Bolivia at 3524.9 per 100,000. Additionally, young women aged 20 to 29 years had a high incidence of cases (13 million), while women aged 35 to 44 years had a higher death rate (3 thousand) [1]. The GBD study also found that between 1990 and 2019, the average prevalence and incidence rates of spontaneous abortions dropped by nearly 56%, while the average and disability-adjusted life years rates dropped by nearly 89% across 21 North African and Middle Eastern countries [2].

An analysis of unintended pregnancies in the MENA region from 2002 to 2022 found that the prevalence was 27%, with Saudi Arabia having the highest rate at 32%. Additionally, unintended pregnancy was linked to both abortion and a history of miscarriages [3]. There are regrettably no data on the incidence of abortion in Jordan due to study restrictions or the delicate nature of the subject.

Pregnancy termination is what both “therapeutic abortion” and “abortion” refer to. The main distinction is the rationale for the procedure: “therapeutic termination” refers to an abortion carried out for a medical cause, whereas “abortion” refers to an abortion carried out at the woman’s request without a medical justification [4]. The topic of abortion has been discussed for many years, generating strong disagreements for and against the procedure [5]. Abortion is a delicate and circumspect topic that is associated with moral, ethical, traditional, and legal issues [6]. The issue is particularly sensitive in the Arab world, where people’s views and opinions regarding abortion, in general, are greatly influenced by a variety of religious doctrines, cultural customs, and legal frameworks [6].

Several complicated rules on abortion exist, and most countries have circumstances that allow safe abortion, including those involving major fetal abnormalities, rape or sexual abuse, risk to life, risk to physical and occasionally mental health, and social and economic considerations [7]. A variety of demographic factors may influence the decision to have a therapeutic pregnancy termination, even though it is completely personal. These factors include women aged 20 to 34, divorced women, women with high educational attainment, women in the workforce, women with multiple children, women residing in both urban and rural areas, women who are members of ethnic minorities, and women with weak religious beliefs [4]. Mental illnesses are more likely in women who undergo a therapeutic pregnancy termination [4,8]; nonetheless, they may resolve with time and health professional assistance [8].

Therapeutic abortion is still controversial and a major concern in many countries. Abortion is legal and typically safe in Canada, where it is also possible to terminate a pregnancy medically or surgically, with public funding frequently available. However, unfettered access to abortion is hampered by obstacles pertaining to time, cost, travel, and regional differences [9]. Abortion is no longer included under the Australian law. However, patients seeking abortion healthcare continue to face obstacles due to the unique regulation of abortion under health law, known as abortion exceptionalism [10]. The USA has implemented new laws that limit access to safe and legal abortion. Abortion access restrictions pose serious risks and harms to health because they increase the likelihood of unfavorable outcomes for mothers and babies, including death [11].

The Freedom House group claims that, abortion is only legally allowed in Jordan to protect a woman’s health or due to fetal impairment [12]. Abortion-related fatwas are typically upheld in Jordan, where the majority of the population is Muslim and has a significant impact on the country’s politics and judicial system. This implies that political decisions and policy-making may use the fatwa as a guide [13].

Most qualitative studies have focused on the experiences of women and/or healthcare professionals with therapeutic abortion and their opinions about medical procedures [14,15,16,17,18]. There is, however, a dearth of qualitative data about the evaluation of public opinion regarding therapeutic abortion. A study that involved semi-structured telephone interviews with 54 US individuals examined their opinions on the safety of different techniques of trying to terminate a pregnancy without medical help. Although they remained concerned about improper use and complications, individuals believed the tablets were safer and more acceptable than other self-managed abortion methods. Compared to facility-based abortion treatment, some thought self-managed abortion could provide more reproductive choice, less stigma, and a safer physical and psychological experience [19].

No qualitative study has been carried out to assess the diverse perspectives Jordanians have on therapeutic abortion. This study fills this gap, in contrast to the limited quantitative research that investigates the occurrence of miscarriages and associated factors [20], as well as the attitudes of health sciences students on abortion in Jordan [21]. The aim of this study was to explore Jordanians’ attitudes toward therapeutic abortion.

## 2. Materials and Methods

### 2.1. Study Design

A qualitative approach was employed to evaluate the various perspectives on therapeutic abortion held by individuals from a variety of demographic backgrounds. Two primary topics were identified in order to explore the opinions of the chosen individuals: (1) opinions regarding therapeutic abortion, and (2) determine who has the authority to make abortion decisions.

### 2.2. Setting and Participants

The study was carried out in Irbid, in the Northern region of Jordan, with a sample of 12 adults who were recruited from various public places. The selection of this city was based on a previous study that demonstrated the availability of post-abortion care and adequately comprehensive abortion care under the law [22]. The only requirements to participate in this study are being a Jordanian citizen and a resident of Irbid city. Purposive sampling is used as the basis for determining the sample size. The participants were recruited using flyers in public places, including markets and mosques. The study was voluntary, and participants were provided with comprehensive information to help them make an informed decision about participating.

Unique codes were provided to the identified participants in order to protect their identities. In light of this, the participants were given the names P1, P2, P3, … P12. This was an essential ethical precaution in order to protect the participants’ identities.

### 2.3. Data Collection

Data were collected using interviews that focused on two key questions: (1) What is your feeling on therapeutic abortion, in the circumstance where such pregnancy poses critical health risk to the mother? (2) In cases when therapeutic abortion is recommended, who do you think needs to make the decision about whether the woman should take it? Additionally, the participant’s age, gender, education level, and marital status were among the basic questions that were asked. The interview guide was created by the first author (RM), who then used an iterative and collaborative process with the other team members (HA, MA, and NA) to receive their input on the questions’ focus, language, and flow. The interview was pilot tested with one non-study sample who did not participate in the study but shared characteristics with study participants. Subsequently, the study team reviewed the interview guide (Table 1) to ascertain whether any modifications were required.

The interviews, which lasted an average of 30 min, were conducted by the first author in a private, confidential environment between April and May 2025. Verbatim transcriptions of the interviews were made in Arabic and cross-checked against the audio recordings. The transcriptions were translated into English to guarantee that the meaning was equal using back-translation techniques with English and Arabic bilingual specialists involved in this study (RM and NA). The interviewer took written notes during the interviews to identify topics that needed additional discussion with participants.

### 2.4. Data Analysis

Thematic analysis was employed to examine the data, as described by Braun and Clark [23]. This process started with verbatim transcription of the responses. The transcripts were cleaned and modified to find and organize codes. Based on the interview guide and the aim of the study, the initial coding was created manually. Key domains, including attitudes regarding therapeutic abortion and determining who should make the decision about performing a therapeutic abortion were reflected in defined codes. The codes were created, and the topics were combined and examined to create themes and sub-themes that resulted in data that were logical, concise, non-repeating, and coherent. As a result, twelve codes were produced from the study. Four main themes and seven sub-themes were created to convey the study’s findings. Data were coded independently by two team members (HA and MA) who had prior experience with qualitative research, and the code list was finalized by comparing and contrasting the data while interpreting them. Disagreements were discussed first between the two researchers and then with the research teams until an agreement was reached. The code book had records of every coding process. The procedure of thematic analysis is illustrated in Table 2.

### 2.5. Reflexivity and Trustworthiness

This study was conducted with reflexivity. The research team allowed for the development of a thoughtful and impartial methodology throughout the data gathering and analysis process. The research team critically considered how their professional responsibilities and disciplinary backgrounds may influence their interpretations during the frequent team meetings that were convened to explore developing codes and themes. The application of trustworthiness was used to guarantee the study findings’ dependability, credibility, conformability, and transferability [24]. The researchers ensured dependability by recording field notes at every stage of the study. Credibility was maintained by verbatim transcription and recording of in-depth interviews in order to assure the accuracy of the data gathered from every participant. Through auditing, conformability was attained to guarantee that themes and sub-themes were coherent and that the outcomes accurately represented the participants’ viewpoints. To ensure transferability, the procedures were described so that the same methodology could be used in other settings.

The first author is a nursing specialist and current faculty member. In addition to being active faculty members, the second and third authors (HA and MA) have extensive experience in qualitative data analysis and community health nursing fields. The fourth author (NA) is a researcher in public health with experience in methodology processes, including data collection and analysis. The reliability of the study was clearly disclosed at every stage of the study. The first three authors were responsible for the data collection and initial coding, which was subsequently verified by the fourth author to reduce any potential bias and increase the reliability of the results. Through this process, themes and sub-themes may be verified, and adding a new perspective to the final analysis. Prior to the analysis, the statements of the participants were directly included in accordance with the verifiability principle. This ensured that the data utilized to support the research findings was transparent. To ensure consistency, the data coding stage was completed independently by the second and third authors. If there are disagreements, the other research team will determine the final code.

### 2.6. Ethical Considerations

Ethical approval was obtained from the Faculty of Nursing Research Ethics Committee at Irbid National University (Ref: IRB0013). The autonomy and respect of participants are among the primary ethical issues taken into account. The first author asked participants if they would be interested in participating in the study in order to gain their consent. This study protected the participants’ privacy and anonymity by not recording any personally identifying information about them. The collected data were protected from unauthorized access.

## 3. Results

### 3.1. Participants’ Characteristics

Table 3 displays the demographic characteristics of the 12 respondents whose data were collected. It is noteworthy that among the responders, there were seven more women (n = 7) than men (n = 5). The majority of responders had completed higher education (n = 9) and were unmarried (n = 7).

### 3.2. Thematic Outcomes

#### 3.2.1. Theme 1: Unconditional Rejection

The theme “unconditional rejection of therapeutic abortion” describes moral and religious customs that oppose giving birth, even when doing so could save the mother’s or the infant’s life or health. Therapeutic abortion is generally prohibited by this perspective, which usually prioritizes the sacredness of potential life as being untouchable. Two sub-themes were developed under this theme: (1) morally and religiously wrong and (2) sanctity of potential life. All participants stated that they frequently believe abortion to be a practice that endangers life and is immoral and spiritually wrong.

According to P3, abortion is viewed as “*illegal and a serious sin (HARAM). It is a termination of life, [which] goes against the teachings of the Quran*”.

P8 expressed “*Abortion is forbidden by our religion, which also declares it to be a sin. We do it to support mothers in need, but it makes us feel horrible about ourselves*”.

Additionally, P12 stated that abortion is not only seen as morally and religiously wrong, but it is also identical to killing.

*“Abortion has to do away with human life, right? Then it means it is a process that goes against our religious, our ethical, our lives, life of the unborn, and can derail life of the mother. It is only through life that we still exist on earth…”*

#### 3.2.2. Theme 2: Conditional Acceptance

The second theme result is the “conditional acceptance” of therapeutic abortion only in extreme circumstances or when the mother’s health is in danger. The responders clarify that only in certain circumstances should therapeutic abortion be permitted. Under this theme, three sub-themes emerged: (1) women at serious risk, (2) infant clinical sign, and (3) medical intervention.

One of the respondents expressed that “*For me, it felt like taking care of a baby is the best thing ever, what happens if the baby dies? how would be logical to lose both lives, when the baby is already in a poor state?*” (P5).

P1 further noted that “*I experienced medical care in my area as being strengthened. In my opinion, doctors who confer with prominent religious leaders can offer guidance on the legality and safety of abortion*”.

P6 also gave explanation about the condition.

*“I would cite one example where a friend of mine got herself into a rape case. She felt devastated, stigmatized. Her mental health deteriorated to extend that she almost committed suicide… how would she even live to raise that child? Some cases are severe”.*

#### 3.2.3. Theme 3: Empathetic and Women-Centered Attitudes

The third theme was formed by the woman’s interest and empathy, which gave rise to a sub-theme “pregnancy’s psychological toll”. P2 pointed out that “*I get the impression that women who are having abortions are extremely eager and desperate to regain their lives. To accompany this, they have been emotional and depressed. One of the most difficult choices they have ever made in their lives is this one*”.

P7 noted “*Abortion is like withdrawing your emotions and going through the motions without enjoying life. The pregnancy would still suffer the deprivation of weak emotional connection if mother does not have the interest of keeping it*”.

Other respondent further stated “*From overall look I can understand what women are thinking. Abortion does bother them and they would feel everlasting stigma when if not interested in keeping such child*” (P10).

#### 3.2.4. Theme 4: Abortion Decision

The second topic was who should be in charge of deciding whether or not the practice should be put into effect. The respondents identified two distinct groups of people—religious leaders and medical professionals—who ought to offer recommendations. The respondents also stated that the individual’s consideration is not necessary to take into account for medical purposes.

P11 stated “*We should be able to rely on our medical specialists to assist with abortion; these are our only option. But I suppose the first obstacle is our own self-confidence*”.

Other responder noted “*When women are threatened with abortion, the doctors’ expertise is crucial. I have faith in them, and I know that everything they say is grounded on their expertise and presented with tact and understanding*” (P9).

P4 also expressed “*If everyone would have the freedom to make a choice when to undertake abortion, then the medical experts and religious authority would get watered down. It would be risky and even the mothers can lose their lives in the process… abortion is a serious issue that cannot be left in the discrete decision of individuals*”.

## 4. Discussion

This study is the first to explore attitudes toward therapeutic abortion from the perspectives of Jordanians who were recruited from different public settings. The study yielded twelve codes clustered into four themes: (i) unconditional rejection of therapeutic abortion; (ii) conditional acceptance in life-threatening or severe cases; (iii) empathetic and woman-centered attitudes; and (iv) abortion decision.

### 4.1. Unconditional Rejection of Therapeutic Abortion

The participants consistently believed abortion to be immoral and against their religious beliefs. Since the Jordanian public claimed the innate religiously imposed beliefs, this conclusion was supported by the fact that spiritual and religious beliefs took precedence over medical needs [25]. This finding is also supported by religious denomination practices in the United States, which aim to stigmatize and condemn women who have had therapeutic abortions [26,27].

The participants noted that there are no provisions for therapeutic abortion under Islamic principles. They took such strong stances because they were committed to moral absolutism and a rigid interpretation of Islamic religious principles. The opinions of the participants were founded on a strict devotion to the moral principles of Islam, which hold that abortion is an immoral activity reserved for unbelievers and should not be tolerated unless it is performed for medical reasons [28]. In other finding, women have expressed theological or spiritual struggle regarding their choices to have therapeutic abortions [26]. The aforementioned absolutism highlights the commitment to cultural, spiritual, or religious customs that are seen to have a significant impact on the therapeutic abortion practice among Jordanians.

The participants shared their opinions that any other kind of abortion is the same as taking a life. A study conducted in New Zealand has demonstrated a strong moral conviction, with worries about purity and the sanctity of life being important factors in defining societal difficulties surrounding therapeutic abortion [29]. Other discussions have emphasized that pregnancy-related issues like fetal or mother health are insufficient justifications for altering the belief and impression that pregnancy termination is wrong, arguing that it is against life itself [30].

### 4.2. Conditional Acceptance in Life Threating or Severe Cases

The participants strongly expressed support for therapeutic abortion where there is possible harm to the mother or the fetus, with regard to conditional approval in severe or life-threatening circumstances. This was supported by the participants’ awareness of the repercussions of endangering the mother’s and the fetus’s lives. The findings are consistent with the framework that permits abortions for women with life-threatening illnesses in Middle East [31,32,33,34].

According to the participants, therapeutic abortion should be permitted if there is sufficient medical evidence of adverse circumstances that could lead to a poor prognosis. The study found a significant compassionate response given the expected burden of medically raising child, even though the termination of the fetus’s life due to abnormal conditions is not as conspicuous as the reason for obtaining a therapeutic abortion when compared to maternal risks [35].

The participants also stated that a qualified medical professional should prescribe therapeutic abortion after consulting with religious authorities. However, this is not the case in most countries where medical professionals only perform therapeutic abortions [36,37].

### 4.3. Empathetic and Women-Centered Attitudes

The results favored therapeutic abortion when the emotional toll was too high. Woman-centered and empathic attitudes are one of the most important elements to take into account in order to prevent a situation in which a woman’s health is threatened by a continued pregnancy [38]. Several studies support this viewpoint when the public considers the potential risks that could impair the mother’s ability to lead a normal life and take into account her mental health [4].

The participants’ capacity to recognize the substantial psychological toll that women endure informs this empathetic approach. This discussion showed that, in addition to the mother’s health and the fetal conditions, additional variables like financial hardship and incest had a substantial impact on how different people perceived and supported therapeutic abortion [39,40]. The results highlight how important it is to support a more holistic approach that prioritizes the mother’s psychological and emotional well-being. An individual’s physical, emotional, psychological, and social well-being are all considered aspects of their health [41]. Thus, not only in Jordan but in all Middle Eastern-led countries, the humanistic approach is viewed as a substitute for absolutism and more strict interpretations of laws governing therapeutic abortion [42].

### 4.4. Abortion Decision

The findings indicated that the qualification for therapeutic abortion is decided by medical professionals and religious leaders. This strategy appears to be repeated in certain Middle Eastern countries where professional advice is valued more highly than individual motives [43]. This indicates that Jordanians have a great deal of faith in religious authorities and medical specialists, particularly when difficult choices need to be made about whether maternal or fetal health issues qualify for therapeutic abortion [44].

Although the results of the study favored a collaborative and consultative process for reaching decisions regarding who should have a therapeutic abortion, they also pointed out that the procedure should be seen as a respectable and health-promoting medical response rather than a personal choice [45]. These results continue to suggest that religious leaders and cultural practices are linked to health, particularly when it comes to medical ethics, despite changing health needs and advances in research and technology [25,46,47].

## 5. Limitations of the Study

One significant limitation is that this study only included participants from one city, making it impossible to extrapolate the results to other Jordanian cities. The small sample size might not adequately represent the range of viewpoints found in the general public, which could result in a narrow understanding of therapeutic abortion.

Since this study only reflects the viewpoints of people who were recruited from public settings, it is unable to represent the perspectives of health professionals and pregnant women. There may be limitations to conducting interviews in public places because these locations might not have offered the same level of intimacy as in-person interviews, which could have impacted the depth of the responses.

The paucity of similar studies makes it difficult to validate the results of this study. It was challenging and time-consuming to translate the interviews from Arabic to English for analysis.

## 6. Conclusions

Jordanians’ views toward therapeutic abortion were found to be centered on mothers, who are the primary target of abortions. The fact that not all women self-identify as “mothers” in relation to abortion is another significant finding. According to a recent study, not all women who undergo adolescent abortions should use the universally assumed “mother” status [48].

This study found that Jordanians’ attitudes about therapeutic abortion were not all the same. One noteworthy result, though, is that the choice to use therapeutic abortion should frequently be left to the judgment of medical experts or religious leaders rather than depending on individual judgment.

The findings demonstrated the participants’ consistent belief that abortion is morally wrong and goes against their religious convictions. Participants strongly supported conditional approval for therapeutic abortion in extreme or life-threatening situations where there may be harm to the mother or the fetus. Therapeutic abortion was preferred when the psychological toll was too great.

## 7. Recommendations

Further studies on the experiences of pregnant women and medical professionals would be necessary to gain a more comprehensive understanding of therapeutic abortion. There is still more research to be performed on how residential areas affect therapeutic abortion, which could help pinpoint the key factors that influence the formulation of public policy.

Despite the consistency of the main themes and sub-themes identified, more research in this area is clearly needed to validate and expand on these findings. Including focus-group interviews might have provided a more complete picture of the socio-cultural aspects of opinions regarding abortion. Mixed-method approaches could be used in future studies to better understand therapeutic abortion among Jordanians.

## Figures and Tables

**Table 1:** Interview guide.

Topical Area	Interview Question	Purpose of the Question
Feeling on therapeutic abortion	What is your feeling on therapeutic abortion, in the circumstance where such pregnancy poses critical health risk to the mother?	Attitudes towards therapeutic abortion
Decision on therapeutic abortion	In cases when therapeutic abortion is recommended, who do you think needs to make the decision about whether the woman should take it?	Therapeutic abortion decision-making
Participant characteristics	What is your age? What is your gender? Are you married or not? What is your highest level of education?	Demographic variables

**Table 2:** Process on thematic data analysis.

Themes	Sub-Themes	Codes
Unconditional rejection	Morally and religiously wrong	Against ethical Against religious Against Quran
	Sanctity of potential life	Termination of life
Conditional acceptance	Women at serious risk	Rape Suicide
	Infant clinical sign	Sickly baby
	Medical intervention	Medical state
Empathetic and woman-centered attitudes	Pregnancy’s psychological toll	Stigma Emotional deprivation
Abortion decision	Medical and religious authorities	Medical experts Religious leaders

**Table 3:** Participants’ characteristics.

Participants (Pseudonyms)	Age	Gender	Marital Status	Education Level
P1	32	Male	Married	Higher
P2	21	Female	Not married	Higher
P3	33	Male	Not married	Higher
P4	25	Female	Married	Secondary
P5	27	Male	Not married	Higher
P6	19	Female	Not married	Higher
P7	22	Female	Married	Higher
P8	29	Female	Not married	Higher
P9	26	Male	Not married	Secondary
P10	26	Female	Married	Higher
P11	30	Male	Married	Basic
P12	36	Female	Not married	Higher

## Data Availability

The data underlying the study’s findings are not publicly accessible due to participant privacy and confidentiality and ethical constraints.
